# Ecological Risk Assessment Related to the Presence and Toxicity of Potentially Toxic Elements in Ashes from Household Furnaces

**DOI:** 10.3390/ijerph19031770

**Published:** 2022-02-04

**Authors:** Alicja Kicińska, Grzegorz Caba, Hubert Serwatka

**Affiliations:** 1 Department of Environmental Protection, AGH University of Science and Technology, Mickiewicza 30 Av., 30-059 Krakow, Poland; caba@agh.edu.pl; 2 I.J. Mączka Invest s.c., 35-231 Rzeszów, Poland; hubertserwatka@gmail.com

**Keywords:** conventional and alternative fuels, municipal waste, trace metals and metalloids, chemical indicators, Risk Assessment Code (RAC, mRAC)

## Abstract

The study material was comprised of 23 samples of ashes generated after the combustion of conventional and alternative fuels combined with selected fractions of municipal waste. The analyses performed involved determining the total concentration of As, Al, Cr, Fe, Ni, and their bioaccessible, ion-exchange, and carbonate-bound fractions. It was found that all of the samples analysed may display an elevated level of susceptibility to the reduction processes, which undoubtedly increases the mobility of trace elements, including the toxic ones. The predominant elements were Al and Fe, whereas considerably lower concentrations were observed for Ni, Cr, and As. The percentage share of the ion-exchange and carbonate-bound fraction ranged from 49% of the total concentration for As to as much as 0.35% in the case of Fe. The calculated Risk Assessment Code index points to a high risk related to the presence of As, medium to low risk related to the presence of Ni and Cr, and low to no risk related to the presence of Fe and Al. The calculated values of the Ecological Risk Index, associated with the combustion of selected municipal waste fractions and low-quality hard coals, combusted individually or in combination with different types of wood, point to a very high ecological risk. This is mainly related to the high concentrations and toxicity of As.

## 1. Introduction

We are currently witnessing a constant increase in the amount of municipal waste generated as a result of the growing production and consumption in all sectors of the economy [1]. Part of this municipal waste is recycled, part is stored at waste landfills, which comprise waste-type-specific installations, and part is combusted in specialist waste incineration plants, which use high-temperature methods like plasma gasification [2,3,4,5]. Numerous countries in the world operate waste-to-energy facilities, although municipal waste incineration remains a socially controversial practice. One of the major problems related to the management of municipal waste is its illegal combustion in household furnaces [6]. A large part of households in Poland (CEE) is still heated with hard coal, which is combined with various types of municipal waste [7]. This practice results in high concentrations of air pollutants, which include PM_2.5_ and PM_10_ particles or sulphur (SO_2_) and nitrogen (NOx) compounds [8,9]. Waste incineration in household furnaces does not bring any significant benefits, not even energy-related ones. When burnt in household furnaces, the material displays a low energy content, in contrast to its combustion in specialist furnaces. Furthermore, installations intended for waste incineration only or furnaces used in the industrial power sector (in power plants or combined heat and power plants—CHP) are equipped with systems that maximally prevent the emissions of pollutants, especially gases and dusts, into the atmosphere [10,11,12,13]. Furnaces in domestic boiler houses do not have dedusting, desulphurisation, or denitrogenation systems.

Another problem would be residues remaining after the combustion of various types of alternative fuels (i.e., coal, wood, peat, and others) combined with different fractions of municipal waste [6,14,15]. It often happens that these residues, in the form of ash, enter the municipal waste stream collected by municipal enterprises. A large part of this material, especially in rural areas, is disposed of onto arable land, despite its negative impact on soils or the groundwater environment [16,17]. Ashes from waste incineration, power, or CHP plants are most often used as various additives to concrete mixes [5,18,19] or stored, having been properly secured as they can easily be air borne by winds. The process of eolic transportation of the finest particulate fractions may have very negative consequences, especially environmental ones, in the form of an adverse impact on the proper development of plants, such as decreasing their assimilation [10,20,21,22].

The estimated number of households heated with solid fuels in Poland is over 3 million, which results in the generation of 2.5 million tons of ashes annually [23]. Ashes from household furnaces are a problematic type of waste due to the fact that not all communes (administrative units responsible for municipal engineering) provide for the special collection of such waste.

An important legal matter related to the management of ashes generated in household furnaces is their classification in accordance with the applicable waste catalogue. Initially, when communes started to collect household ashes, the latter were classified in the same group as waste generated by power plants or other power-related facilities. However, under the currently applicable legal regulations [24], ashes from household furnaces are classified as municipal waste. The reason for this change was the properties and chemical composition of ashes vary greatly and depend on the type of fuel or fuel mixes, as well as other additives burnt in household furnaces [6,11,12,25,26]. Additives such as municipal waste significantly determine the composition and properties of the ash generated. The main components of ashes are silicon oxide (SiO_2_), iron oxide (Fe_2_O_3_), and aluminum oxide (Al_2_O_3_). The structure of the ash obtained depends mainly on the primary material used and the combustion temperature. Ashes are predominantly fine particulate products [20,27]. However, they are characterised by a certain degree of size variability, making their fraction classification quite broad, from a psammite fraction to fine-grained pelite particulates. The size of ash particles is immensely important when assessing their impact on the plant and soil environment [10,22,26]. As demonstrated in previous studies, the sand and silt fractions are of major importance, as they determine the dustiness and filtrating properties of ashes [20,28,29]. Another important property of ashes is their solubility and high susceptibility of their components, especially the toxic ones, to leaching [11,15,27,30,31]. The latter contain potentially toxic elements (PTEs), which mainly include heavy metals (i.e., Al, Cd, Cr, Fe, Ni, Pb, and Zn) and metalloids (As). Their excessive amounts may be toxic, or even carcinogenic, to living organisms. At the biochemical level, PTEs in excessive concentrations compete with basic metabolites, disrupt the process of major ion exchange, damage cellular membranes, and react with phosphate groups in ADP and ATP. This pertains to all three trophic levels: producers, consumers, and decomposers [26,27].

Given the above-mentioned facts, this paper presents an assessment of the environmental risk related to the presence of Potentially Toxic Elements (PTEs: Al, As, Cr, Fe, and Ni) in ashes generated from the combustion of conventional and alternative fuels combined with various fractions of municipal waste in household furnaces. Based on the mixes prepared, the following parameters were determined:

(i) the total content of PTEs in ashes,
(ii) the amount of PTEs that were bioaccessible to producers,
(iii) PTE fractions present in the material analysed,
(iv) environmental risk (Risk Assessment Code—RAC—and modified Risk Assessment Code—*m*RAC) and Ecological Risk index (ERI) related to the PTEs’ susceptibility of being easily leached from ashes.

The novel aspect to this study is the parametrisation of the assessment of the environmental risk stemming from the presence of PTEs in ashes and their improper handling, such as their deposition on arable land or storage at municipal waste landfills unfit for this type of waste.

## 2. Materials and Methods

The study material was comprised of 23 samples of ashes generated as a result of the co-incineration of primary material mixes. The latter were composed of conventional fuels (C), alternative fuels (AF), and selected fractions of municipal waste (MW) collected from the grate of a Keller 10 kW furnace (approx. 80% AFUE efficiency, maximum working pressure 0.25 MPa). The primary material mixes were selected in a way that most accurately reflected the fuels and additives used in household furnaces.

The furnace charge was comprised of fuel mixes with an addition of single or mixed fractions of municipal waste (Table 1). Prior to being placed in the furnace, the primary material was fragmented (about 2–5 cm fragments) to facilitate combustion. The wood used in the experiment was freshly cut.

The primary material was incinerated in April 2019 under identical conditions for each lot. The hearth and ash collector were thoroughly cleaned after combusting each portion of the material. The exact procedure of obtaining ash samples has been described previously [31].

The 23 ash samples were divided into 5 groups (Table 1):

− group I (AF, *n* = 7) was comprised of alternative fuels from biomass. This group included such materials as: wood of acacia (*Acacia* Mill.), wood of ash (*Fraxinus*), wood of black elderberry (*Sambucus nigra* L.), wood of willows (*Salix* L.), wood of acacia + elderberry + ash + willows, straw, and nuts.
− group II (C, *n* = 5) was comprised of conventional fuels, whose chemical composition was dominated by carbon. This group included 3 types of hard coal: hard coal no. 1, hard coal no. 2, hard coal no. 3, and coal pellets. The three types of hard coal were obtained from different coal storage sites located in various regions of Poland. Coal no. 1 was sourced from Małopolskie Province, coal no. 2 was purchased from a coal storage site in Mazowieckie Province, and coal no. 3 was obtained from Podkarpackie Province. This group also included peat due to the high content of C > 50 wt.%.
− group III (AF + C, *n* = 3) was comprised of alternative fuels mixed with conventional fuels. This group included samples of hard coal no. 1 mixed with wood: ash, willow, and acacia.
− group IV (MW, *n* = 5) was comprised of 5 types of municipal waste fractions. This group included such types of waste as: paper and cardboard, plywood, sawdust, plastic-coated paper cartons, and used disposable diapers.
− group V (C + MW, *n* = 3) was comprised of material obtained after incinerating hard coal mixed with municipal waste. This group included ashes generated from combusting hard coal no. 1 combined with textiles, mixed municipal waste, and plastic.

The exact procedure of ash sample preparation has been described by Kicińska and Caba [31]. The fuels were incinerated according to the following steps:

− a portion of the primary material was placed in the furnace chamber;
− the material underwent incomplete combustion, initiated with a gas burner;
− the furnace and ash were cooled down;
− the ash was collected into a container;
− the coarse fraction was separated using a sieve;
− a sample of the ash was collected and placed in a tightly sealed plastic bag;
− the chamber and bottom hopper were cleaned mechanically and with compressed air before the incineration of the next portion of fuel.

The primary material cobusted and cooled down naturally. In each experiment, the air supply was the same. In the case of mixes, the fuel and waste materials were used in equal proportions, 1:1 *v*/*v*. The incineration conditions were to reflect those found in household furnaces. As most of household furnaces do not feature any built-in measurement system, the furnace used for the experiments was not equipped with one either.

### 2.1. Physicochemical Analyses 

In the preliminary stage of laboratory testing, each ash sample was dried at 105 °C for 2 h to determine the constant weight, and then secured against moisture.

#### 2.1.1. pH

The pH of the aqueous extracts of ashes was analysed in accordance with the [32] PN-EN 12176:2004 standard, using a pH meter (CPC-401), which is an electronic voltmeter indicating the pH of a solution based on the electromotive force measurement. The pH measurement was repeated three times for each extract (1:2.5 solid phase/solution ratio), with the suspension being previously stirred with a glass rod. Each time, the pH electrode was rinsed with distilled water, and any excessive liquid was removed with blotting paper. The results obtained were averaged.

#### 2.1.2. Pseudo Total Content of PTEs

A single-step extraction was conducted in a concentrated mixture of acids (HCl:HNO_3_) at a 1:3 ratio, with the solid phase/solution ratio of 1:10. This concentrated mixture of acids breaks only some of the chemical bonds (silicate bonds may remain intact). Thus, this method is associated with a certain degree of underestimation. However, in light of the research goals of the present study and the fact that substances with a stronger effect are not found in the environment, the authors decided to use this procedure to assess the total content of PTEs. It is more accurate when determining low PTE concentrations, which was of significant importance for the environmental risk assessment. Given that aluminosilicate forms do not dissolve in the solvent used, this kind of degradation is called pseudo total content (TC). Mineralisation was conducted for 2 h in a microwave oven (SCP SCIENCE, type DigiPREP HT) at 130 °C, and then the solutions were cooled down and decanted. 

#### 2.1.3. Ion-Exchange and Carbonate-Bound Fraction—Extraction with CH_3_COOH

To calculate the environmental risk, the first step of the BCR (European Community Bureau of References) extraction was performed [10,33]. The diversity of physicochemical bonds present in the materials studied results in an increase or a decrease in the mobility of elements and can also affect their bioaccessibility. To perform the extraction, a 1 g portion was weighed out from each of the previously dried samples and then placed in PCV test tubes. Next, 40 mL of 0.11 M CH3CCOH was added to each of the tubes. The obtained suspensions were mixed for 16 h at 22 °C. The eluates were then decanted and the concentrations of the elements analysed were determined. The extraction with 0.11 M CH_3_COOH allowed for calculating the percentage share of the exchange fraction easily soluble in acid (F1) relative to the total concentration of PTEs. The procedure provided metals contained at the exchange positions as well as those bound to carbonates.

#### 2.1.4. Bioaccessible Fraction—Extraction with CaCl_2_

To determine the bioaccessible fraction of PTEs, extraction with a 0.01 M CaCl_2_ solution was performed [34]. This solvent was used due to its ionic strength, which is similar to the mean concentration of salts in soils. The suspensions were shaken for 2 h at 22 °C, at a 1:20 solid phase/solution ratio. The eluates were centrifuged, decanted, and filtered.

PTE concentrations (Al, As, Cr, Fe, and Ni) in the solutions analysed (aqueous, exchange-carbonate, and bioaccessible fractions) were determined in an accredited hydrogeochemical laboratory (certificate of accreditation PCA no. AB1050) at AGH University of Science and Technology. PTE measurement precision was 10%, and accuracy ranged from 95% to 109%. The control system of the analyses (QA/QC) was compliant with the PN-EN ISO 17025 standard. In each series of determinations, blank samples, duplicate samples (min. 25%), and marked samples were used. Moreover, the Standard Reference Material (NIST) no. 1633b (Trace Elements in Coal Fly Ash) was analysed, for which the differences in all the element concentrations did not exceed 4%. 

The results were statistically compiled using Excel 2013 and Statistica ver.13.1 software.

### 2.2. Environmental and Ecological Risk (RAC, mRAC, ERI)

The environmental risk related to the presence of PTEs in ashes from household furnaces was calculated using the Risk Assessment Code (RAC). This method involves comparing the percentage of cations found in ion-exchange positions and bound to carbonates (F1 BCR fraction), with their total concentration in a given sample [35]. RAC was calculated using Formula (1):

(1) $$\mathrm{RAC}=\frac{\;amount\;of\;cations\;of\;each\;element\;bound\;with\;ion\;positions\;and\;carbonates}{total\;content\;of\;element}\;\times \;100\%$$

Values between 1–10% denoted low risk, 11–30%—medium risk, 31–50%—high risk, and over 50%—very high risk [6,36].

Another index analysed was the modified Risk Assessment Code (*m*RAC), calculated based on formulas suggested by Håkanson [37], which additionally includes m—the “toxic-response” factor. The calculated RAC value is multiplied by Tri—the toxic-response factor for a given substance (Tr for As equals 10, for Ni—5, and for Cr—2). 

The last tool used in the study was the Ecological Risk Index (ERI), which represented the risk from the sum of metals contained in the study material analysed [37]. It is the sum of the calculated mRAC values (in this case for As, Cr, and Ni).

## 3. Results and Discussion 

### 3.1. Physicochemical Characteristics of Ashes

#### 3.1.1. pH

The pH values for all the ashes analysed ranged between 8.89 and 15.07, with a mean of 11.98 (Table 2). These results clearly indicate an alkaline pH for the study material.

The highest pH values were observed for ash samples generated from the combustion of hard coal no. 1 with the addition of plastic (pH = 15.07). A similarly high pH was found in the aqueous extract of the same coal type mixed with ash wood (pH = 14.74). The lowest pH value, on the other hand, was recorded for the ash sample from hard coal no. 3 (pH = 8.89). The mean values calculated for individual groups of the primary material can be arranged in the following descending order: mixes of alternative fuels and coal (13.58) > mixes of municipal waste and coal (12.99) > alternative fuels (12.55) > municipal waste (11.19) > coals (9.58).

The pH values obtained corresponded with the findings published by other authors [11,14,15]. The pH value closely determines the physicochemical changes that may occur in the environment due to ash deposition. The larger the deviation from the value of 7 in the pH scale, the greater the changes in the mobility and release of elements in environmental processes, which has been demonstrated in a paper by Kicińska and colleagues [38].

#### 3.1.2. Pseudo Total Content of PTEs in Ashes

**Figure 1:**
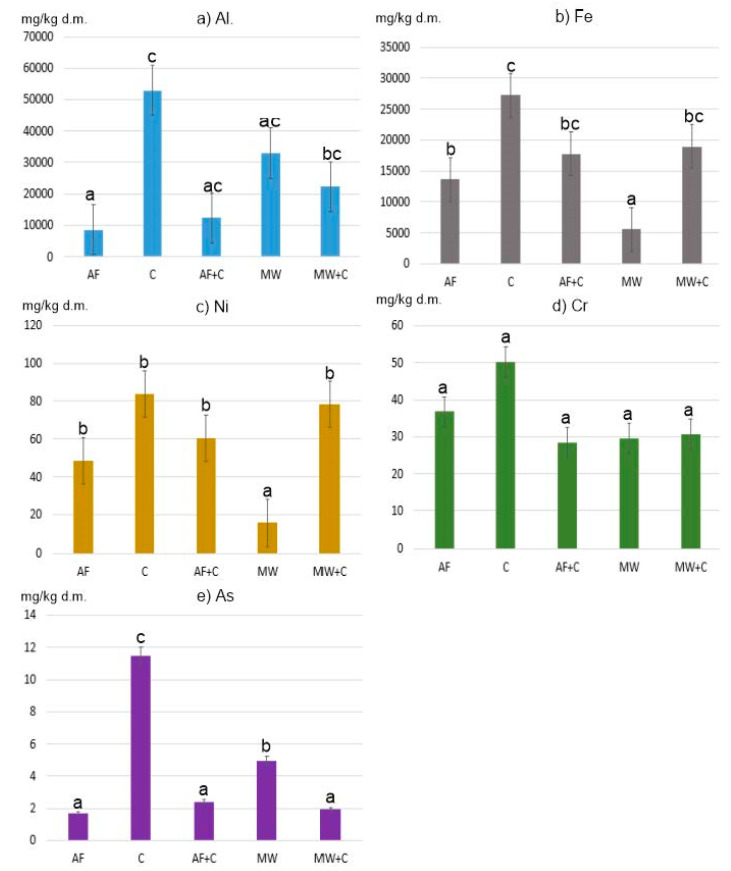
Average content of PTEs in ashes: (**a**) Al, (**b**) Fe, (**c**) Ni, (**d**) Cr and (**e**) As. Means marked with the same letter do not differ at *p* = 0.05.

The measured pseudo total content of PTEs (Al, As, Cr, Fe, and Ni) in ashes varied considerably and was as follows (min.–max., in mg/kg d.m.): 1039–111,388, 0.29–24.28, 3.71–82.10, 2300–33,725, and 4.57–144.17, respectively (Table 2). Elements found in the highest amounts in the ashes studied were Fe and Al (Figure 1). 

**Figure 2:**
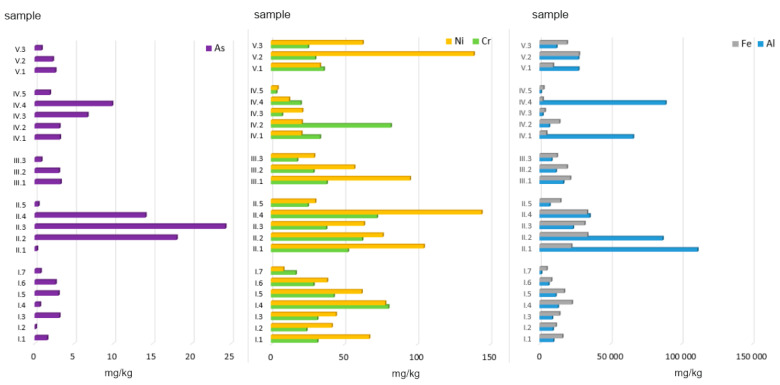
Total concentration of PTEs in ashes burned in a household furnace.

The highest Fe content was found in hard coal no. 2 and no. 3 (respectively: 33,725 mg/kg and 31,759 mg/kg), and in coal pellets (33,529 mg/kg). The mean Fe content in alternative fuel samples (i.e., various types of wood, straw, and nuts) incinerated alone or in combination with coal was 13,612 and 17,757 mg/kg, respectively. The lowest Fe content was observed in ashes generated from combusting various municipal waste fractions, namely paper, cardboard, sawdust, plastic-coated paper cartons, and disposable diapers. These values did not exceed 5000 mg Fe/kg (Figure 2). The only exception was plywood, whose ash sample contained 14,052 mg Fe/kg. High Fe content (137,000 mg/kg d.m.) was observed by Huber et al. in municipal solid waste incineration bottom ash [15].

Ashes obtained after combusting coals and selected municipal waste fractions (and a mix of these two groups) displayed the highest content of Al (in mg/kg d.m.): 52,879, 32,994, and 22,280, respectively. In the coal group, the highest concentration of this element (111,388 mg/kg) was found in ash generated from hard coal no. 1. Ashes obtained from the other types of coal (no. 2 and no. 3) also displayed high concentrations of Al, namely 86,813 and 23,642 mg/kg, respectively. These values are in line with the results obtained by Kalembasa et al. [39], who reported that ashes generated from hard coal and lignite combustion in the power industry contained 9505–41,061 mg Al/kg.

Our study demonstrated statistically significant differences (at *p* = 0.05) between Al and Fe concentrations in individual ash samples from the groups of alternative fuels, coals, and municipal waste (Figure 1).

In all of the samples studied, the determined concentrations of Ni, Cr, and As were clearly lower (Figure 2). 

The mean Ni content in ashes generated from coal combustion was 83.88 mg/kg, and that was the highest mean value for this element. The lowest mean value of 16.09 mg/kg was recorded for the group of ashes obtained from municipal waste combustion. The highest Ni concentration was found in ashes from coal pellets (144.17 mg/kg), and the lowest in ashes from diapers (4.57 mg/kg). The differences in the content of Ni between municipal waste ashes and all the other groups were statistically significant (Figure 2).

As for Cr, the differences in the content of this element between all the samples analysed were not statistically significant (Figure 1). However, it is worth mentioning that the highest mean value (50.21 mg/kg d.m.) observed for ashes generated from conventional fuel combustion (coals) was nearly twice as high as the lowest mean value for the group of ashes obtained from municipal waste combustion (29.51 mg/kg d.m.). In the group of ashes generated from the combustion of various municipal waste fractions (group MW), the highest Cr content was observed for plywood (82.10 mg/kg d.m.), and the lowest for diapers (3.71 mg/kg d.m.). Such a high Cr content in the plywood analysed may be due to the various types of glues used, which contain polyacetates, vinyls, aliphatic resins, and polyurethane.

The last element analysed was As. The lowest concentration of As (data in mg/kg d.m.) was found in ashes from the combustion of alternative fuels (1.72) and the mix of hard coals with those fuels (2.41), and with municipal waste (1.94). On the other hand, the highest concentration of As was observed for ashes from hard coal no. 3 (24.28). High As content was also noted for ashes from coal pellets (14.10) and hard coal no. 2 (18.07). As concentrations in ashes obtained from conventional fuel combustion differed statistically significantly from those observed for other ash groups (Figure 2).

Cr, Ni, and Fe content determined in ashes from hard coal and peat was comparable to the values obtained by Kalembasa et al. [39]. These authors reported the following mean values for ash samples from conventional fuel combustion (hard coal and lignite): 29.2, 75.98, and 12,993 mg/kg d.m, respectively, which is in line with our results. On the other hand, Zang et al. [17,40], who analysed Ni, Cu, Zn, Cd, Sn, and Pb concentrations in ashes from the combustion of municipal waste, demonstrated that the values were 10–200 times higher as compared to the concentrations found in soils. This considerably affects the ashes’ potential for agricultural use, as they pose a risk to the soil environment, which has been reported in studies by Horák et al. and Kuboňova et al. [30,41].

To conclude, the mean PTE content calculated for all 23 ash samples can be arranged in the following decreasing order (in mg/kg d.m.):

Al (26,680) > Fe (16,408) >> Ni (55.41) > Cr (36.57) >> As (4.75).

#### 3.1.3. Ion-Exchange and Carbonate Fraction

Ash extraction with 0.11 M acetic acid allowed for the determination of the absolute amount and the share of the acid-soluble fraction (comprising water- and acid-soluble forms) and ions found in exchange positions (Table 3).

**Figure 3:**
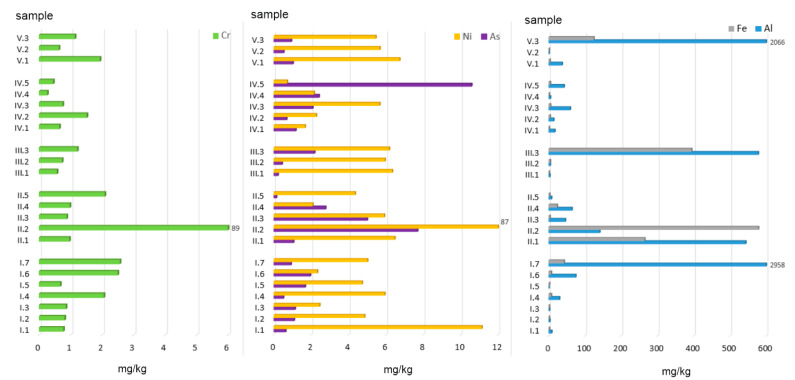
Ion- and carbonate-exchange fractions of PTEs in ashes burned in a household furnace.

Al proved to be most susceptible of being dissolved in an acidic environment (in terms of the absolute amount). The mean amount of extracted Al cations calculated for all the samples was 304.91 mg/kg, which is 2.05% of the total concentration (Figure 3). The lowest susceptibility was observed for As, whose mean content in all the extracts analysed was 1.93 mg/kg, i.e., 49.41% of the pseudo total content.

The highest amount of Al was extracted from alternative fuels, especially ashes obtained from the combustion of walnuts (958 mg/kg) and the mix of hard coal no. 1 with plastic packaging (2065 mg/kg). High quantities of ion-exchange Al, namely 543 and 578 mg/kg, were also observed for ashes from hard coal (no. 1 and no. 2) and hard coal mixed with acacia wood, respectively (Figure 3). For all the other samples, Al content did not exceed 100 mg/kg. Mean Al concentrations calculated for individual groups (as a % of the total concentration, %TC) can be arranged in the following order: AF (5.10) > MW + C (3.15) > AF + C (1.59) > C (0.30) > MW (0.08).

The amount of Al found in the ion-exchange and carbonate-bound fraction in the material analysed was considerably lower and ranged between 0.15 and 10.59 mg/kg. The highest amount of Al was extracted from ashes obtained from diaper (10.59 mg/kg) and hard coal combustion (5.01–7.70 mg/kg for various types of coal). However, the conversion of the values to a percentage share in the total concentration revealed that Al was the most leachable element (data in %TC): MW (68) > AF (66) > MW + C (43) > AF + C (40) > C (29).

Regarding Cr, the amounts extracted ranged from 0.27 to 88.89 mg/kg, with the highest concentration found in ashes obtained from hard coal no. 2 and the lowest in those from plastic-coated paper cartons (Figure 3). The content of Cr in F1—ion-exchange and carbonate-bound fractions in individual ash groups can be arranged in the following order (data in %TC): C (37) > MW + C = AF (4) > AF + C (3) > MW (2.5).

Fe displayed highly diverse concentrations with regard to its absolute and relative amounts extracted with acetic acid from the material analysed. The highest amounts of this element in the exchange and carbonate fraction were found in ashes obtained from the combustion of hard coals (265.02–578.76 mg/kg) and coals mixed with acacia wood (394 mg/kg) and with plastic packaging (124 mg/kg). With regard to the mean Fe content expressed as a percentage share in the total concentration, the material analysed can be arranged in the following order (data in %TC): AF + C (0.75) > C (0.64) > MW + C (0.23) > AF (0.07) > MW (0.06).

The last element analysed was Ni. Its amount extracted in F1 BCR ranged from 0.72 to 86.90 mg/kg, with the highest values observed for ashes obtained from hard coal no. 2 (86.90 mg/kg) and acacia wood (11.13 mg/kg). The lowest Ni content (0.72 mg/kg) was found in ashes from diapers. When calculated as a percentage share in the total concentration, the highest amounts of Ni were extracted from (data in %TC): C (25) > MW (15) > AF (11) > AF + C (10) > MW + C (8).

The mean concentrations of individual elements analysed in the eluates of all 23 ash samples form the following order (in mg/kg): Al (304.91) > Fe (72.88) > Ni (8.19) > Cr (4.61) > As (1.93). When calculated as a percentage share in the total concentration, the values for *n* = 23 can be arranged as follows (data in %TC): As (49) >> Ni (14) > Cr (10) > Al (2) > Fe (0.3).

#### 3.1.4. Bioaccessibility of PTEs

The 0.01 M CaCl_2_ extraction test developed by Houba et al. [34] allows for the extraction of metal forms directly accessible to plants and is particularly helpful in soil quality assessment. This extraction solution is also used to assess the risk of shallow groundwater contamination with the mineral elements considered in the present study.

The greatest variability was observed for Al, whose amounts extracted with 0.01 M CaCl_2_ ranged from 0.46 to 6910.45 mg/kg. The highest Al doses accessible to plants were found in ashes from (data in mg/kg): plastic-coated paper cartons (6910), paper and cardboard (880), and peat (206). The highest concentration of bioaccessible Al (1581 mg/kg) was observed for the municipal waste group (MW). As for the other groups, the following mean Al concentrations were obtained (data in mg/kg): AF + C (102) > C (89) > AF (29) > MW + C (26).

**Figure 4:**
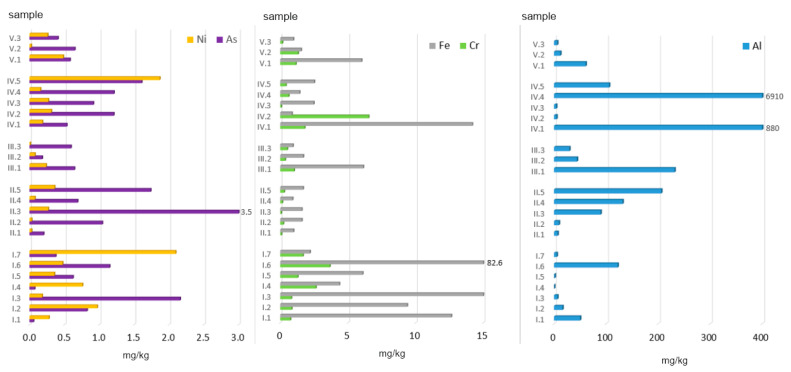
Bioaccessible fraction of PTEs in ashes burned in a household furnace.

In the case of As, the highest bioaccessible amounts were found in ashes from hard coal no. 3 (3.53 mg/kg) and black elder wood (2.16 mg/kg). The highest mean amount of bioaccessible As (1.44 mg/kg) was observed for the coal group (Figure 4). As for the other groups, the following mean values were calculated (data in mg/kg): MW (1.10) > AF (0.75) > MW + C (0.55) > AF + C (0.47).

The amount of bioaccessible Cr in the ashes analysed ranged from 0.07 to 6.52 mg/kg. Its mean content (in all the samples) was 1.05 mg/kg, i.e., about 3% of the total concentration. The highest amount of bioaccessible Cr was found in ashes obtained from the combustion of plywood (6.52 mg/kg) and straw (3.66 mg/kg). With regard to all of the groups analysed, the following mean values were calculated (data in mg/kg): MW (1.89) > AF (1.67) > MW + C (0.88) > AF + C (0.64) > C (0.17).

The next element, Fe, displayed the lowest bioaccessibility (Figure 4), which on average was 0.05% of its total concentration. In terms of absolute values, the observed range was 0.86–82.65 mg/kg in the eluates analysed. The highest amount of Fe was found in ashes from straw. Other high values were recorded for ashes from black elder wood (15.16 mg/kg), paper and cardboard (14.18 mg/kg), and acacia wood (12.64 mg/kg), and it was the alternative fuel group that presented the highest mean concentration of bioaccessible Fe. The mean values obtained can be arranged in the following order: (AF) 18.93 mg/kg > MW (4.29) > AF + C (2.92) > MW + C (2.84) > C (1.35).

The last of the metals analysed was Ni, which also presented a very low level of bioaccessibility. Its mean content of 0.36 mg/kg constituted only 1% of this element’s total concentration. The mean amounts of bioaccessible Ni for individual ash groups form the following decreasing order (in mg/kg): AF (0.73) > MW (0.56) > MW + C (0.26) > C (0.15) > AF + C (0.11). The highest concentrations of bioaccessible Ni were found in ashes from walnuts (2.10 mg/kg) and diapers (1.87 mg/kg). The most important factors affecting the bioaccessibility of elements to plants include: the total concentration of potentially bioaccessible elements in soil, and the concentration of elements in the soil solution and their mutual quantitative proportions. A significant role is also played by the mobility of elements from the solid soil phase to the liquid phase, and then their absorption by roots and transport to the aerial parts of plants. In a study by Karwowska and Dąbrowska [42], the bioavailable fraction of Ni in municipal sewage sludge was about 20 mg/kg on average, which is a significantly higher value than that obtained in the present analysis.

To conclude, the mean concentrations of PTEs extracted with a 0.01 M solution of calcium chloride, calculated for all the ash samples, form the following decreasing order (data in mg/kg): Al (365.40) >> Fe (6.07) > Cr (1.05) > As (0.86) > Ni (0.36). These values correspond to: 1.25, 0.05, 3.27, 25.24, and 1.13 %TC, respectively (Table 4).

The bioaccessible fraction of PTEs does not necessarily correspond to their content in organisms. There is a number of factors affecting the varying resistance of a given species or even single individuals to high PTE bioaccessibility [43]. The most important ones include: individual traits (health condition, ways of adaptation), hormonal balance, age, and genetic features. Furthermore, each organism has a certain capacity for biological accumulation. When it is exceeded, passive absorption and toxic effects occur [35]. 

### 3.2. Environmental and Ecological Risk Assessment

#### 3.2.1. Risk Assessment Code (RAC)

The environmental risk related to the chemical composition of ashes was determined using the Risk Assessment Code (RAC), which involves calculating the ratio between the fraction that is easily soluble in an acidic environment (F1 BCR) and the total concentration of the elements analysed. In the case of this method, the environmental risk is classified into one of five risk groups (Table 5). For the ash groups analysed, means and medians were calculated for individual RAC values obtained for the 23 samples studied.

The highest RAC values were observed for As (14.76–68.25), denoting medium risk (for ashes obtained from coals) through high risk (for ashes from the mix of alternative fuels and municipal waste with coals) to very high risk (for ashes from alternative fuels and municipal waste).

High environmental risk (RAC = 37) was also found for Cr present in ashes from the coal group. Considerably lower RAC scores (Av: 7.60–15.54) were obtained for Ni, denoting medium environmental risk. Another element analysed was Cr, with RAC scores falling within a 2.48–4.04 range, which indicates low risk. As mentioned above, the exception was the coal group, which presented high environmental risk. Low risk was also found for Al with respect to ashes obtained from alternative fuels (RAC = 5.10), municipal waste mixed with coal (RAC = 3.15), and alternative fuels mixed with coal (RAC = 1.59). No risk related to the presence of Al was observed for the other two groups, namely ashes obtained from coal and municipal waste combustion. A lack of environmental risk was also demonstrated for Fe, with the corresponding RAC scores ranging between 0.06 and 0.75.

If RAC: 

The mean values (arithmetic mean and median) for all the ashes studied formed the following decreasing order of environmental risk scores related to the presence of the elements analysed in ashes (RAC—arithmetic mean): As (40.46) >> Ni (12.03) > Cr (6.48) > Al. (1.07) > Fe (0.19). The differences in the mean values (Av and Me) were not considerable and essentially did not affect the environmental risk group assigned.

The above analysis indicates that the presence of Al and Fe in ashes does not entail any environmental risk. Nevertheless, the authors of the present study decided that further analysis would include the level of toxicity (T_ri_) presented by individual elements, especially those particularly hazardous due to their carcinogenic properties (As, Cr, and Ni).

#### 3.2.2. Modified RAC (mRAC)

The modified RAC (*m*RAC) was calculated in the study. This is an index of potential ecological risk [44,45,46], reflecting the varying toxicity level of As, Ni, and Cr, which is: 10, 5, and 2, respectively (Table 6). The authors believe that *m*RAC is considerably more significant and valid in environmental analyses than RAC, as it more clearly presents the differences between the samples analysed.

Very high potential environmental risk (*m*RAC ≥ 320) related to the presence of As in ashes was observed for: acacia, black elder, and willow wood, and the multi-species wood mix and straw, nuts, coal no. 1, coal no. 2, coal mixed with acacia wood, paper and cardboard, diapers, and hard coal mixed with textiles. Considerable or moderate potential ecological risk was observed for the other ash samples. In the case of Ni, very high ecological risk was found only for the sample of ashes generated from the combustion of hard coal no. 2. For the other samples, the potential ecological risk ranged from low to considerable. With regard to the *m*RAC (<40) calculated for Cr, the samples presented low potential ecological risk, except for one. This sample was generated from the combustion of hard coal no. 2, and its *m*RAC score was 284, which indicates high potential ecological risk.

#### 3.2.3. Ecological Risk Index (ERI)

The cumulative Ecological Risk Index (ERI) calculated for all the ashes analysed indicated:

− very high ecological risk (ERI ≥ 600) in the case of ashes from: diapers (5568), hard coal no. 1 (3675), hard coal no. 1 mixed with acacia wood (2720), hard coal no. 2 (1278), hard coal no. 1 mixed with plastic packaging (1133), willow wood (817), and straw (784);
− considerable ecological risk (300 ≤ ERI < 600) in the case of ashes from: multi-species wood mix (599), hard coal no. 1 and textiles (505), acacia wood (490), sawdust (463), paper and cardboard (413), black elder wood (397), peat (395), and plastic-coated paper cartons (335);
− moderate ecological risk (150 ≤ ERI < 300) in the case of ashes from: plywood (277), hard coal no. 1 and municipal waste mix (258), hard coal no. 3 (257), coal pellets (207), and hard coal no. 1 and willow wood (204);
− low ecological risk (ERI < 150) in the case of ashes from: ash wood (142) and hard coal no. 1 and acacia wood (106).

## 4. Conclusions

Based on the study material and the analyses conducted, it was found that:

1) All the ash samples analysed have alkaline pH and may be more susceptible to reduction processes, which will undoubtedly increase the mobility of trace elements, including the toxic ones;
2) The predominant elements in the composition of ashes were Al (Av for *n* = 23, 26,680 mg/kg) and Fe (16,408 mg/kg). Considerably lower concentrations were observed for Ni, Cr, and As: 55, 36, and 4.7 mg/kg, respectively. The highest concentrations of Al, As, Fe, and Ni were found in ashes obtained from hard coal combustion. The highest Cr concentration, on the other hand, was displayed by the ash sample obtained from plywood;
3) The percentage share of the ion-exchange and carbonate-bound fraction (F1 BCR) of the cations analysed ranged from 49% of the total concentration for As, through 14% (Ni), 10% (Cr), and 2% (Al), to 0.35% in the case of Fe, which is an unfavourable outcome from the environmental and toxicological perspective. The highest amounts of Cr, Fe, and Ni easily soluble in an acidic environment were found in ashes generated from coal combustion. Ashes obtained from a municipal waste mix combined with hard coal presented the highest share of easily extracted Al, and ashes from municipal waste and hard coals had the highest amount of As extracted in F1 BCR;
4) The amounts of bioaccessible PTEs corresponded with their total concentrations. For all the samples analysed, their mean values were as follows (data in mg/kg): Al (365.40) >> Fe (6.07) > Cr (1.05) > As (0.86) > Ni (0.36). The highest amount of bioaccessible Fe and Ni was found in ashes from alternative fuels, Al and Cr in ashes from municipal waste, and As in ashes from various types of hard coal;
5) The highest environmental risk (high risk) was related to the presence of As in the ashes studied. This mainly pertains to ashes obtained from alternative fuels, municipal waste, coals, and mixes of these materials. Medium risk to low risk was related to the presence of Ni and Cr in all the material groups analysed. Low risk to no risk related to the presence of Fe and Al was found for all the ash groups studied;
6) Very high ecological risk stems from the deposition of ashes obtained from the combustion of selected fractions of municipal waste (i.e., diapers, textiles, paper, and cardboard) and low-quality hard coals, combusted individually or in combination with different types of wood. This is related to high concentrations and toxicity of As.

## Figures and Tables

**Table 1:** Primary materials burned in household furnaces.

Group of Ashes	No. of Sample	Primary Burned Material	*n*
I–AF	I.1.	wood of acacia (*Acacia* Mill.)	7
	I.2.	wood of ash (*Fraxinus*)	
	I.3.	wood of black elderberry (*Sambucus nigra* L.)	
	I.4.	wood of willows (*Salix* L.)	
	I.5.	wood of: acacia + elderberry + ash + willows	
	I.6.	straw	
	I.7.	nuts	
II–C	II.1.	coal 1	5
	II.2.	coal 2	
	II.3.	coal 3	
	II.4.	coal pellets (ekogroszek)	
	II.5.	peat	
III–AF + C	III.1.	coal 1 + wood of ash	3
	III.2.	coal 1 + wood of willows	
	III.3.	coal 1 + wood of acacia	
IV–MW	IV.1.	paper and cardboard	5
	IV.2.	plywood	
	IV.3.	sawdust	
	IV.4.	plastic-coated paper cartons	
	IV.5.	diapers	
V–MW + C	V.1.	coal 1 + textiles	3
	V.2.	coal 1 + mix of municipal waste	
	V.3.	coal 1 + PET drink bottle	

AF—alternative fuels based on biomass, C—coal, MW—municipal waste; *n*—number of samples.

**Table 2:** pH and total concentration of PTEs in household ashes.

Group of Primary Materials	Parameters	pH *_H2O_	Al	As	Cr	Fe	Ni
			(mg/kg d.m.)				
AF	Min.–Max.	11.82–14.50	1286–13,045	0.68–3.14	16.99–80.42	5077–22,894	8.51–78.22
	Av. ± SD	12.55 **	8616 ± 3548	1.72 ± 1.14	36.79 ± 19.26	13,612 ± 5499	48.65 ± 21.33
C	Min.–Max.	8.89–13.39	7167–111,388	0.29–24.28	25.30–72.57	14,752–33,725	30.36–144.17
	Av. ± SD	9.58	52,879 ± 39,560	11.44 ± 9.59	50.21 ± 16.91	27,249 ± 7491	83.88 ± 38.45
AF + C	Min.–Max.	13.23–14.74	8496–16,800	0.84–3.30	18.00–38.25	12,291–21,652	29.60–95.14
	Av. ± SD	13.58	12,287 ± 3428	2.41 ± 1.11	28.48 ± 8.28	17,757 ± 3979	60.59 ± 26.87
MW	Min.–Max.	10.49–13.79	1039–88,879	1.94–9.86	3.71–82.10	2300–14,052	4.57–21.44
	Av. ± SD	11.19	32,994 ± 37,060	4.98 ± 2.92	29.51 ± 28.31	5557 ± 4333	16.09 ± 6.68
MW + C	Min.–Max.	12.52–15.07	11,988–27,453	0.87–2.63	25.44–36.23	9534–27,846	33.55–138.85
	Av. ± SD	12.99	22,280 ± 7278	1.94 ± 0.76	30.68 ± 4.41	18,903 ± 7482	78.41 ± 44.38
for *n* = 23	Min.–Max.	8.89–15.07	1039–111,388	0.29–24.28	3.71–82.10	2300–33,725	4.57–144.17
	Av. ± SD	11.98	26,680 ± 31,012	4.75 ± 6.19	36.57 ± 21.03	16,408 ± 9514	55.41 ± 38.35

d.m., dry mass; AF, alternative fuels based on biomass; C, coals; MW, municipal waste; Av., arithmetical average; * pH values are averaged for 3 measurements; ** means calculated according to Kicińska et al. [38].

**Table 3:** Content of ion- and carbonate-exchange fractions of PTEs in household ashes.

Group of Ashes	Parameters	Al	As	Cr	Fe	Ni
		(mg/kg d.m.)				
AF	Min.–Max.	0.76–958.25	0.52–1.96	0.68–2.57	1.67–42.88	2.33–11.13
	Av. ± SD	439.69 ± 1028.49	1.14 ± 0.49	1.47 ± 0.81	9.30 ± 13.91	5.21 ± 2.72
	(% of TC)	(5.10)	(66.55)	(3.99)	(0.07)	(10.71)
C	Min.–Max.	7.95–543.42	0.15–7.70	0.88–88.89	3.09–578.76	2.09–86.90
	Av. ± SD	160.65 ± 196.24	3.34 ± 2.74	18.76 ± 35.07	174.88 ± 224.88	21.15 ± 32.91
	(% of TC)	(0.30)	(29.18)	(37.37)	(0.64)	(25.21)
AF + C	Min.–Max.	3.41–578.17	0.23–2.19	0.57–1.22	1.51–394.35	5.95–6.34
	Av. ± SD	195.32 ± 270.72	0.96 ± 0.88	0.84 ± 0.28	133.58 ± 184.40	6.16 ± 0.16
	(% of TC)	(1.59)	(39.73)	(2.96)	(0.75)	(10.16)
MW	Min.–Max.	4.95–59.58	0.69–10.59	0.27–1.52	1.22–5.06	0.72–5.67
	Av. ± SD	27.63 ± 20.27	3.40 ± 3.65	0.73 ± 0.43	3.37 ± 1.69	2.50 ± 1.68
	(% of TC)	(0.08)	(68.25)	(2.48)	(0.06)	(15.54)
MW + C	Min.–Max.	0.91–2065.63	0.54–1.04	0.64–1.94	1.47–124.43	5.46–6.73
	Av. ± SD	701.25 ± 964.88	0.84 ± 0.22	1.24 ± 0.54	43.25 ± 57.41	5.96 ± 0.56
	(% of TC)	(3.15)	(43.33)	(4.04)	(0.23)	(7.60)
for all samples *n* = 23	Min.–Max.	0.76–958.25	0.15–10.59	0.27–88.89	1.22–578.76	0.72–86.90
	Av. ± SD	304.91	1.93	4.61	72.88	8.19
	(% of TC)	(2.05)	(49.41)	(10.17)	(0.35)	(13.84)

AF, alternative fuels based on biomass; C, coals; MW, municipal waste.

**Table 4:** Bioavailable fraction of PTEs in household ashes.

Group of Ashes	Parameters	Al	As	Cr	Fe	Ni
		(mg/kg d.m.)				
AF	min.–max.	0.46–122.27	0.06–2.16	0.75–3.66	2.20–82.65	0.18–2.10
	av. ± SD	28.94 ± 41.31	0.75 ± 0.68	1.67 ± 1.02	18.93 ± 26.36	0.73 ± 0.61
	(% of TC)	(0.34)	(43.82)	(4.53)	(0.14)	(1.50)
C	min.–max.	6.97–206.20	0.20–3.53	0.07–0.29	0.91–1.69	0.03–0.36
	av. ± SD	88.79 ± 75.65	1.44 ± 1.16	0.17 ± 0.08	1.35 ± 0.33	0.15 ± 0.14
	(% of TC)	(0.17)	(12.59)	(0.33)	(0.00)	(0.18)
AF + C	min.–max.	29.56–231.64	0.18–0.64	0.36–1.03	0.93–6.13	0.01–0.24
	av. ± SD	101.78 ± 92.02	0.47 ± 0.21	0.64 ± 0.28	2.92 ± 2.29	0.11 ± 0.10
	(% of TC)	(0.83)	(19.65)	(2.24)	(0.02)	(0.18)
MW	min.–max.	4.25–6910.45	0.53–1.61	0.08–6.52	0.86–14.18	0.16–1.87
	av. ± SD	1581.10 ± 2684.80	1.10 ± 0.36	1.89 ± 2.39	4.29 ± 4.99	0.56 ± 0.66
	(% of TC)	(4.79)	(22.02)	(6.40)	(0.08)	(3.47)
MW + C	min.–max.	6.24–60.81	0.41–0.65	0.16–1.32	0.97–6.01	0.02–0.48
	av. ± SD	26.42 ± 24.44	0.55 ± 0.10	0.88 ± 0.51	2.84 ± 2.25	0.26 ± 0.19
	(% of TC)	(0.12)	(28.15)	(2.86)	(0.02)	(0.33)
for all samples *n* = 23	min.–max.	0.46–6910.45	0.06–3.53	0.07–6.52	0.86–82.65	0.01–2.10
	av.	365.40	0.86	1.05	6.07	0.36
	(% of TC)	1.25	25.24	3.27	0.05	1.13

**Table 5:** Risk assessment code (RAC) values calculated for household ashes.

Group of Ashes		RAC				
		Al	As	Cr	Fe	Ni
AF	Av.	5.10	66.55	3.99	0.07	10.71
	Me	0.09	53.67	2.70	0.01	7.62
C	Av.	0.30	29.18	37.37	0.64	25.21
	Me	0.16	18.69	2.33	0.07	9.28
AF + C	Av.	1.59	39.73	2.96	0.75	10.16
	Me	0.04	14.67	2.52	0.03	10.43
MW	Av.	0.08	68.25	2.48	0.06	15.54
	Me	0.01	31.12	1.94	0.02	15.09
MW + C	Av.	3.15	43.33	4.04	0.23	7.60
	Me	0.14	39.40	4.50	0.04	8.69
For all samples (*n* = 23)	Av.	1.07	40.46	6.48	0.19	12.03
	Me	0.15	39.57	2.83	0.07	10.30

If RAC: 
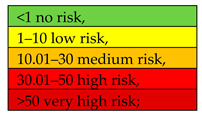

**Table 6:** Modified Risk Assessment Code (*m*RAC) and potential Ecological Risk Index (ERI) calculated for household ashes.

Group of Ahses	No.	*m*RAC *				ERI **
		Primary Burned Material	As	Cr	Ni	
			T_r*i*_ (10)	T_r*i*_ (2)	T_r*i*_ (5)	
AF	I.1	wood of acacia (Ac)	**402.4**	4.9	82.7	490.0
	I.2	wood of ash (A)	77.0	6.7	58.4	142.1
	I.3	wood of black elderberry (Be)	**364.3**	5.4	27.6	397.3
	I.4	wood of willows (W)	**773.7**	5.1	38.0	***816.8***
	I.5	wood of: Ac + Be + A + W	**557.4**	3.2	38.1	598.6
	I.6	straw	**736.7**	17.2	30.4	***784.3***
	I.7	nuts	**1234.1**	30.3	294.7	***1559.0***
C	II.1	coal 1	**3640.8**	3.7	30.9	***3675.4***
	II.2	coal 2	**426.1**	284.4	**567.7**	***1278.2***
	II.3	coal 3	206.2	4.7	46.4	257.3
	II.4	coal pellets (ekogroszek)	196.9	2.7	7.2	206.8
	II.5	peat	306.9	16.5	71.7	395.2
AF + C	III.1	coal 1 + wood of ash	69.9	3.0	33.3	106.2
	III.2	coal 1 + wood of willows	146.7	5.0	52.2	203.9
	III.3	coal 1 + wood of acacia	**2602.2**	13.6	104.4	***2720.2***
MW	IV.1	paper and cardboard	**368.8**	3.9	40.1	412.8
	IV.2	plywood	219.6	3.7	53.9	277.2
	IV.3	sawdust	311.2	19.7	132.2	463.2
	IV.4	plastic-coated paper cartons	246.0	2.6	86.8	335.4
	IV.5	diapers	**5464.9**	24.7	79.0	***5568.5***
MW + C	V.1	coal 1 + textiles	**394.0**	10.7	100.3	505.0
	V.2	coal 1 + mix of MW	233.3	4.2	20.4	257.9
	V.3	coal 1 + PET drink bottle	**1080.2**	9.0	43.4	***1132.6***

Tri, toxic-response factor; If: * mRAC < 40 low potential ecological risk, 40 ≤ *m*RAC < 80 moderate potential ecological risk, 80 ≤ *m*RAC < 160 considerable potential ecological risk, 160 ≤ *m*RAC < 320 high potential ecological risk, *m*RAC ≥ 320 very high ecological risk (**bolded**), ** If: ERI < 150 low ecological risk, 150 ≤ ERI <300 moderate ecological risk, 300 ≤ ERI <600 considerable ecological risk, ERI ≥ 600 very high ecological risk (***bolded*** and ***italic***).

## Data Availability

Original data included in this publication will be made available upon request after a second companion publication is available.
